# Boswellic acid sensitizes gastric cancer cells to Cisplatin-induced apoptosis via p53-mediated pathway

**DOI:** 10.1186/s40360-020-00442-1

**Published:** 2020-09-01

**Authors:** Shadia Al-Bahlani, Ikram A. Burney, Buthaina Al-Dhahli, Safiya Al-Kharusi, Fakhra Al-Kharousi, Amani Al-Kalbani, Ikhlas Ahmed

**Affiliations:** 1 Department of Allied Health Sciences, College of Medicine and Health Sciences, Sultan Qaboos University, P. O. Box 35, PC 123 AlKhoud, Muscat, Oman; 2 Department of Medicine, College of Medicine and Health Sciences, Sultan Qaboos University, Muscat, Oman; 3 Department of Pharmacology and Clinical Pharmacy, College of Medicine and Health Sciences, Sultan Qaboos University, Muscat, Oman

**Keywords:** P53, CDDP, AKBA, Apoptosis and gastric cancer

## Abstract

**Background:**

Cisplatin (CDDP) is an effective anticancer drug for Gastric cancer (GC) that induces apoptosis by altering pro- (p53) and anti-apoptotic (Akt and NFkB) proteins; however, chemoresistance remains a big challenge. Additional compounds with promising anticancer effects such as AKBA (Acetyl-keto-beta boswellic acid) may overcome the resistance. However, its role in CDDP-induced apoptosis in GC has not been studied. This study aimed to examine the effectiveness of AKBA on p53-mediated, CDDP-induced apoptosis in GC cells. AGS and NCI-N87 cells were treated with different concentrations (0, 25, 50, 100 μM) of CDDP and/or AKBA.

**Methods:**

P53, Akt and NFkB proteins and apoptosis were assessed by Western blot and flow cytometry. The role of p53 was determined by inhibiting its function via the siRNA approach.

**Results:**

The results revealed that CDDP and AKBA significantly increased p53 content in both cells, while Akt and NFkB were significantly decreased. Both compounds significantly induced apoptosis in a dose-dependent manner. AKBA sensitized GC cells to CDDP-induced apoptosis by altering the protein expression. P53 downregulation affected Akt and NFkB proteins with a slight increase in apoptosis induction in the combination treated groups.

**Conclusions:**

Altogether, our findings suggest that AKBA enhances GC cell sensitivity to CDDP-induced apoptosis via the p53 pathway.

## Background

Gastric cancer (GC) is the fourth most common cancer and the 3rd most common cause of cancer-related mortality worldwide [1, 2]. Its incidence varies with geographical regions, for example, the incidence is 20 times higher in Japan, Chile and Costa Rica compared to North America and the Northern Europe [3]. GC is one of the 10 most common cancers in Oman [4]. Cisplatin (Cis–diammine dichloro platinumor; CDDP) and its derivatives remain the standard of care in the treatment of GC. CDDP causes damage to the cancer cells by exerting cytotoxic effects on DNA replication through crosslinking, eventually resulting in apoptosis [5, 6]. Although CDDP is effective initially, eventually tumors develop resistance due to an increase in DNA repair or elevated levels of glutathione, which neutralizes the reactive oxygen species formed by CDDP [7].

CDDP induces apoptosis by activating the p53-mediated apoptotic pathway. Factors that activate p53 protein include internal and external stress signals [8]. In normal situations, p53 binds to Mdm2 protein preventing its function as a transcription factor, but when mutated, expression of p53 is up-regulated [9]. Other proteins are also involved in regulation of cell survival and death and adversely affect p53-mediated apoptosis such as Akt and NFkB proteins [10]. Akt is considered to be one of the most important molecular drivers inducing malignancies, including the GC. Activation of Akt results in cell proliferation and survival through activation of other substrates such as mTOR and Cyclin D. CDDP induces Akt cleavage and thus apoptosis [11, 12]. Nuclear factor kappa-light-chain-enhancer of activated B cells (Rel/NFkB) is also a transcription factor that binds to an inhibitory molecule called IKB. CDDP induces IKB degradation and hence activation of NFkB [13].

AKBA (Acetyl-keto- boswellic acid), extracted from frankincense of *Boswella Sarca* tree, has shown promising anticancer effects in certain types of cancer including brain, colon, prostate and pancreas [14, 15]. Previously published reports have shown that the effect was mediated by activation of several apoptosis pathways including the PI3K/Akt, the Wnt/β-catenin, and the NFkB/COX-2 signaling pathways [16].

Liu J and his team demonstrated that AKBA can induce apoptosis in colon cancer cells; however, its role in p53-dependent-apoptosis is not studied yet [17]. In addition, the combined effect of AKBA and CDDP on GC cells and the mechanism of cytotoxicity has not been studied yet. Therefore, this study was designed to explore the role of p53 in both CDDP- and AKBA- mediated apoptosis and whether, there is a synergistic effect in overcoming the chemoresistance.

## Methods

### Reagents and antibodies

F-12 K Nut Mix medium, Roswell Park Memorial Institute medium (RPMI), 0.25% Trypsin–EDTA, penicillin and streptomycin and fetal bovine serum (FBS) were obtained from Gibco (Scotland, UK). CDDP (50 mg/50 mL) was obtained from Hospira, USA. AKBA (10 mM) was obtained from SIGMA-ALDRICH (Saint Louis, USA). FlowCellect Annexin Red Kit was obtained from Millipore (Darmstadt, Germany). Primary antibodies of rabbit monoclonal anti -Akt, −NFkB,-GAPDH, & -PARP and mouse monoclenal anti-p53 in addition to secondary antibodies (Gout anti-rabbit and -mouse IgG (HRP conjugated) were obtained from cell signaling technology (Beverly, MA, USA). P53 siRNA was also purchased from the same company. SuperSignal West Dura Extended Duration Substrate was obtained from Pierce (Rockford, IL, USA).

### Cell culture

Human gastric cancer cell lines NCI-N87 and AGS were purchased from ATCC (Virginia, USA) and have been recently reported by Liu et al. [18] Both cell lines were cultured at 37 °C in a humidified 5% carbon dioxide atmosphere. Seeding density for AGS was 8000 cells /mm while for NCI-N87 was 15000 cells/mm in an appropriate 24-well plate or 6-well plate. RPMI-1640 was used as media for NCI-N87, and was supplemented with 10% FBS, 1% penicillin and 1% streptomycin, while F-12 K media (Gibco, UK) was used for AGS cells with the same supplements. Both types of cells were treated with different concentrations of CDDP (0, 50 and 100 μM) and/or AKBA (0, 25, 50, and 100 μM). These concentrations were used previously in similar studies on gastric, colon and pancreas cell lines [17–22]. Finally, they were harvested after 24 h for further analysis.

### P53 siRNA transfection

In NCI-N87 cell line, p53 protein expression was down-regulated using p53-siRNA, as described earlier [22]. Briefly, cells were treated for 24 h with 150 nM of p53 siRNA, followed by CDDP and/or AKBA treatment for another 24 h. Cells were then harvested for further analysis.

### Protein extraction and Western blot analysis

Protein extraction and Western Blot were performed as described previously [22]. Briefly, AGS and NCI-N87 cell lines were harvested and treated with lysis buffer (50 mM Hepes, 150 mM NaCl, 1 mM EGTA, 10 mM Sodium Pyrophosphate (Nappi), 1.5 mM MgCl_2_,100 mM NaF, 10% Glycerol, 1% Triton X-100) to extract the proteins. Samples were then centrifuged to purify the proteins, and BCA protein assay kit was used to quantify the amount of proteins (BioVision, California, USA).

Western blot was done following BioRad protocol. Fifty microgram of protein samples were loaded and ran on 10% SDS-PAGE gel. Proteins were transferred to a Polyvinylidene difluoride PVDF membrane (Thermo Scientific, Rockford, lL, USA), which was blocked using 5% milk for 30 min. Next, primary antibody (11000) in 5% milk to the membrane was added, and incubated for an hour at room temperature. Then the membrane was washed 3 times with 1x TBST to remove any non-specific binding. Secondary IgG (15000) was added to the membrane and incubated for an hour at room temperature; the membrane was washed 3 times again with 1x TBST. Protein was then detected using West Dura kit (Pierce, Rockford, IL, USA). The solution was prepared by mixing (1) of the stable peroxide buffer and Luminal/ Enhancer solution. The membrane was incubated for 3 min at room temperature. Eventually G-Box machine using GeneSys software was used to detect the bands.

### Apoptosis analysis

FlowCellect Annexin Red Kit was used to detect vaible and apoptotic cancer cells. First, cell samples were prepared in 1X Assay Buffer HSC; 100 μL of each cell sample was pipetted into an appropriate well and 100 μL Annexin Red Working solution was added. Cells were incubated at 37 °C for 15 min. Following incubation, each well was washed once with 200 μL 1X Assay Buffer HSC. Afterwards the cell pellet was resuspended in 195 μL 1X Assay Buffer HSC and 5 μL of 7-AAD were added per well. Then, wells were incubated at room temperature for 5 min in the dark. Finally, the analysis was done on dual laser flow cytometer.

### Statistical analysis

All data are expressed as mean ± standard error (SE) of at least three independent replicates. They were analyzed by the single-factor analysis of variance (One-way ANOVA) using the SPSS program®, version 23. Significant differences between measurements are indicated at ***P < 0.001, **P < 0.05 and *P < 0.01. Bands of Western Blot were quantified using ImageJ Software.

## Results

### CDDP had differential effect on proteins expression and induced apoptosis in GC cells

**Fig. 1:**
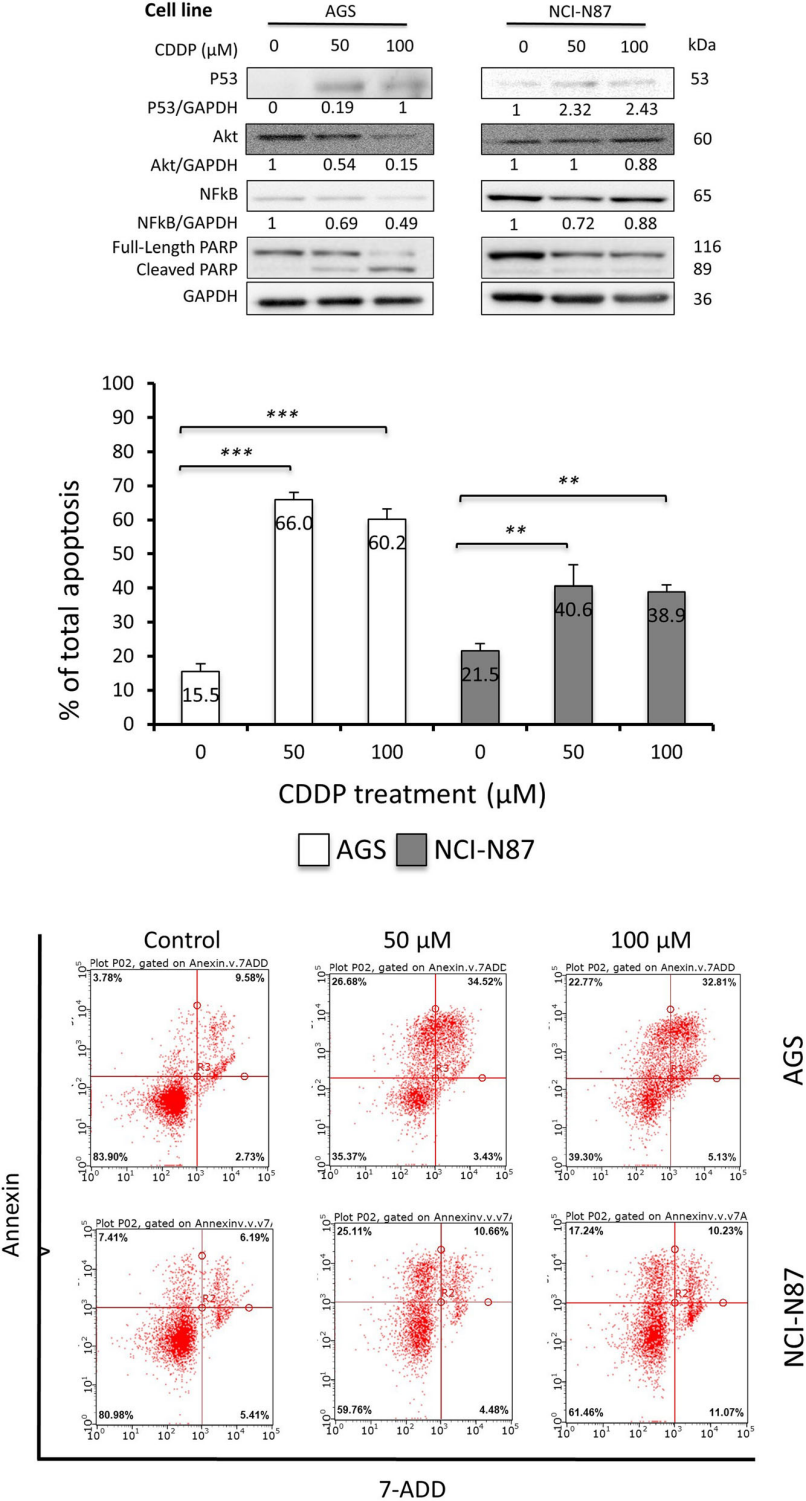
CDDP had differential effect on proteins expression and induced apoptosis in GC cells. **a** Western blot shows the effect of CDDP on protein expression in AGS and NCI - N87 cell lines. The expression of tumor suppressor protein 53 increased with CDDP concentration, whilst Akt and NFkB expressions reduced, notice the cleaved PARP which indicates apoptosis **b** Graphical presentation of apoptosis percentages in AGS and NCI-N87 cell lines treated with different concentrations of CDDP showing significant increase in apoptosis in comparison to control concentration (*P < 0.05, **P < 0.01, ***P < 0.001). The response of AGS cells is more evident than NCI-N87. **c** Flow cytometry graphs showing the effect of different doses of CDDP on AGS and NCI-N87 cells, each graph shows alive cells, early and late apoptotic cells, and dead cells

**Fig. 2:**
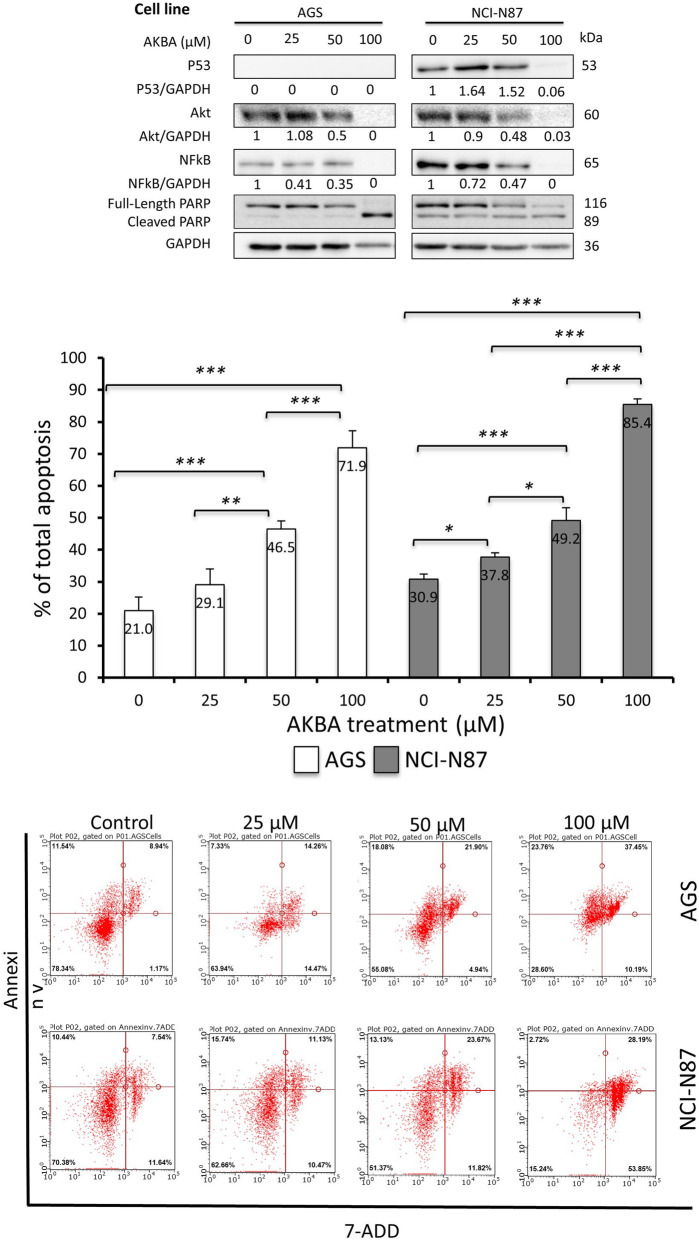
AKBA exhibited similar effect as CDDP on proteins expression and apoptosis in GC cells except for p53. **a** Western blot shows the effect of AKBA on protein expression in AGS and NCI - N87 cell lines. The expression followed a similar pattern to the cells treated with CDDP **b** Graphical presentation of apoptosis percentage in AGS and NCI-N87 cell lines treated with different concentrations of AKBA showing significant increase in apoptosis in comparison to control concentration (*P < 0.05, **P < 0.01, ***P < 0.001). The concentration of 100 μM was found to be highly toxic and the apoptosis percentages reached 71.9 and 85.4% in AGS and NCI-N87 cell lines respectively. **c** Flow cytometry graphs showing the effect of different doses of AKBA on AGS and NCI-N87 cells. Each graph shows alive cells, early and late apoptotic cells, and dead cells

We first aimed to measure the effect of CDDP on p53, Akt and NFkB proteins expression and apoptosis induction in GC cells. Fig. 1a reveals a significant increase in the expression of p53 in NCI-N87 and AGS cells while Akt and NFkB expression is significantly decreased. AGS cells show minimal expression of only p53 protein in the control group. PARP cleavage was a good indicator of the cells response to CDDP treatment. Total-PARP expression decreased in parallel with an increase of it’s cleaved form, indicating its activity in facilitating apoptosis. GAPDH was used as a loading control to normalize to total protein content the expression of proteins of interest. Figure 1b & 2c show that CDDP significantly induced apoptosis in a concentration-dependent manner in both cell lines; AGS cells responded more compared to the NCI-N87 cells.

### AKBA exhibited similar effect as CDDP on proteins expression and apoptosis in GC cells except for p53

Although some studies have shown that AKBA may induce apoptosis in certain cancer types, none examined its effect in gastric cancers and on the p53-depedent pathway. Therefore, after measuring the effect of CDDP, we examined if AKBA will also exhibit similar effect on the protein expression and apoptosis induction in the studied cells. Unlike CDDP, AKBA had no effect on p53 protein in AGS cells, but increased its expression in NCI-N87 cells at a concentration of 25 and 50 μM. On the other hand, it had an effect similar to CDDP in reducing Akt and NFkB proteins expression in both cell lines (Fig. 2a). AKBA had a similar effect in inducing apoptosis in both cell lines as indicated in Fig. 2b and c.

A dose of 100 μM of AKBA was found to be toxic as indicated by the loss of protein expression and high percentage of apoptosis (71.9 and 85.4% in AGS and NCI-N87 cell lines respectively), suggesting its behavior as a potent cytotoxic compound.

### AKBA sensitized GC cells to CDDP-induced apoptosis via altering proteins expression

**Fig. 3:**
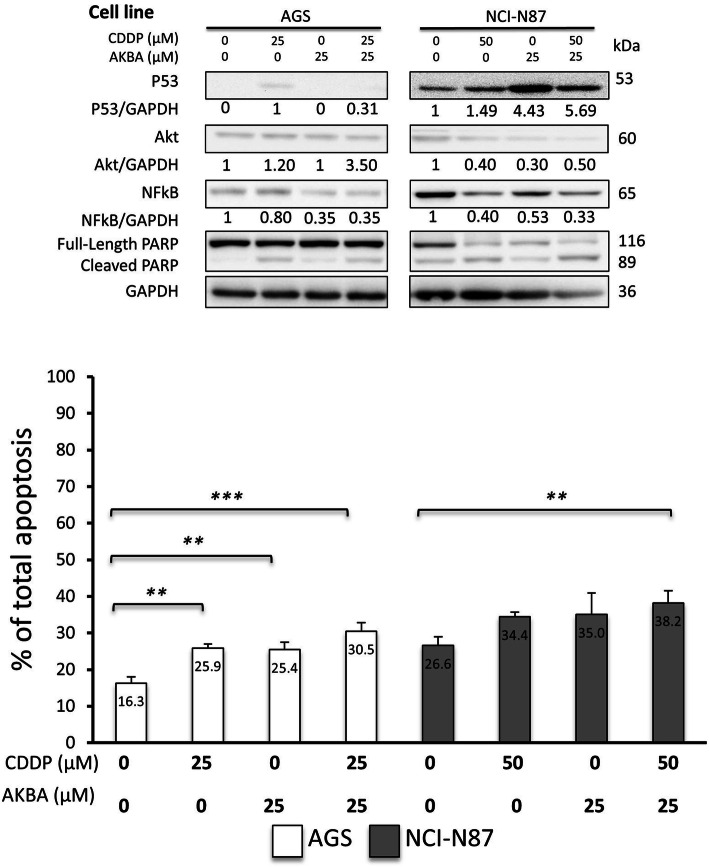
AKBA sensitized GC cells to CDDP-induced apoptosis via altering proteins expression. **a** Western blot shows the effect of combined CDDP and AKBA on protein expression in AGS and NCI - N87 cell lines. **b** Graphical presentation of apoptosis percentage in AGS and NCI-N87 cell lines treated with different concentrations of CDDP and AKBA showing significant increase in apoptosis in comparison to control concentration (*P < 0.05, **P < 0.01, ***P < 0.001)

We investigated the extent to which the combination of AKBA and CDDP were associated with modification of apoptosis and protein expression of p53, Akt, and NFkB in GC cells. Based on Fig. 1b and 2b, optimal concentration of each compound and for each cell lines were selected; NCI-N87 cells were treated with 50 μM of CDDP and 25 μM of AKBA, while AGS cells were treated with 25 μM each of CDDP and AKBA. The optimum concentrations were selected based on our findings from Figs. 1 and 2 in which these concentrations showed significant difference compared to the controlled group. In addition, Liu et al and his team supported our selection when they found that IC_50_ of CDDP for NCI-N87 cell line is higher than AGS cells [18], hence NCI-N87 cells were treated with 50 μM and AGS 25 μM. The combined treatment of both CDDP and AKBA showed more effect on the expression of three proteins compared to the single treatment in both cells as shown in Fig. 3a. However, the same was not true for apoptosis induction, in which combining CDDP and AKBA had no significant increase compared to the single treatment, yet it was significant compared to the control group for AGS and NCI-N87 cells. Altogether, this suggested that both compounds have additive effect that work on similar apoptotic pathways.

### P53-dependent pathway mediated CDDP- and AKBA-induced apoptosis in GC cells

**Fig. 4:**
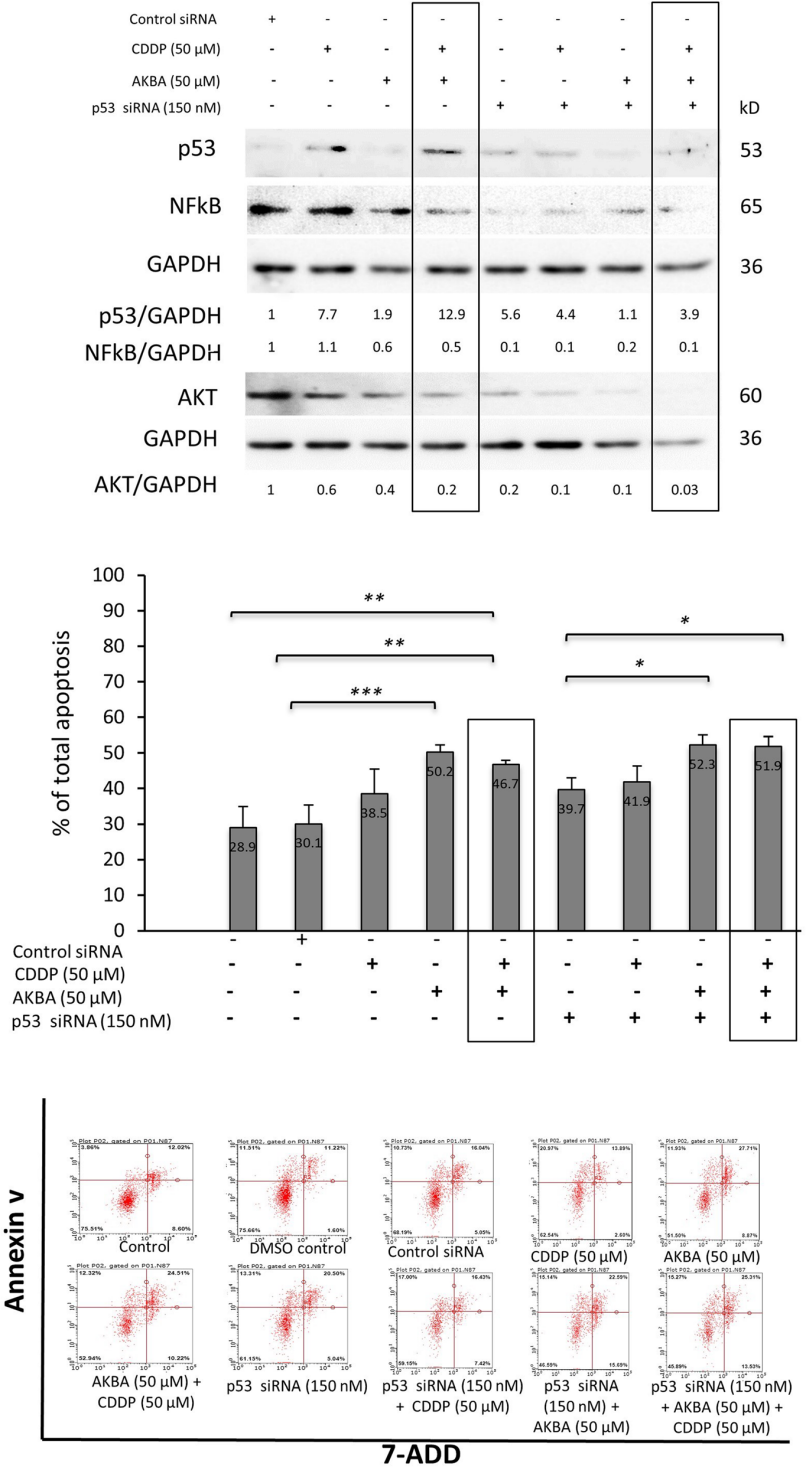
P53-dependent pathway mediated CDDP- and AKBA-induced apoptosis in GC **a** Western blot shows the effect of siRNA, CDDP and AKBA on protein expression in NCI - N87 cell line. P53 siRNA was successful in reducing the increase in p53 expression post treatment of CDDP and AKBA up to approximately 70%. The expression of Akt and NFkB proteins was further reduced in the combined treatment when p53 protein was down-regulated **b** Graphical presentation of apoptosis percentage in NCI-N87 cell line treated with different treatments showing varying degrees in apoptosis in comparison to control concentration. Apoptosis induction showed a slight trend of increase with no significant difference between treated groups with and without p53 down regulation. (*P < 0.05, **P < 0.01, ***P < 0.001). **(c)** Flow cytometry graphs showing the effect of different treatments on NCI-N87 cells. Each graph shows alive cells, early and late apoptotic cells, and dead cells

Although we showed that both CDDP and AKBA influence p53 protein expression, its role in regulating these cells sensitivity to both compounds had not been studied yet. Therefore, to assess such a specific role of CDDP- and AKBA-induced apoptosis, p53 protein was down-regulated using siRNA in NCI-N87 cells only, since it was not detected in AGS cells at the basal level. Figure 4a shows consistent effect of both compounds on the expression of all three proteins; as well on apoptosis induction in both cells. P53 siRNA was successful in reducing the increase in p53 expression post-treatment up to approximately 70%. The expression of Akt and NFkB proteins was further reduced by the combined treatment when p53 protein was down-regulated, suggesting their role as downstream effectors in the p53 pathway. Although, apoptosis induction showed a slight increase in these cells, there was no significant difference between the treated groups, with and without p53 down regulation, suggesting that p53 pathway might contribute to both CDDP-and AKBA-dependent apoptosis at the upstream mediators.

## Discussion

Understanding the mechanisim of action of CDDP and AKBA in apoptosis induction in GC cells is a crucial step to suggest successful treatment strategy. This study demonstrated an important role of the p53 pathway in the regulation of GC sensitivity to CDDP- and AKBA-dependent apoptosis. It also provides evidence that a defect in such pathway might result in resistance to CDDP and/or AKBA treatment. As many drugs that affect multiple pathways, boswellic acid acetate was also found to induce apoptosis via p53-independent pathway as Xia L et al and his team showed in myeloid leukemia cell [23]. They demonstrated that the levels of apoptosis-related proteins Bcl-2, Bax, and Bcl-XL were not modulated by boswellic acid acetate, however; it induced Bid cleavage and decreased mitochondrial membrane potential without production of hydrogen peroxide. By using general caspase inhibitor (Z-VAD-FMK) and a specific caspase-8 inhibitor II (Z-IETD-FMK), boswellic acid acetate–induced apoptosis was blocked, suggesting that such mechanism was through activation of caspase-8 and not p53. Such variation in boswellic acid acetate action might be due to the differences in genetic background of both cancer types as well as the activities of these tested proteins.

Here, we have shown that both CDDP and AKBA significantly increased the expression of p53 in NCI-N87 and AGS cells while Akt and NFkB expressions were significantly decreased. The two compounds also induced significant apoptosis in a dose-dependent manner in both cell lines in which AGS cells responded more effectively compared to the NCI-N87 cells. Furthermore, AKBA sensitized GC cells to CDDP-induced apoptosis via altering the protein expression. Gene manipulation of p53 expression by siRNA, and thus protein reduction, affected expression of Akt and NFkB proteins and thus increased apoptosis in the group treated by a combination of CDDP and AKBA.

Several studies have confirmed the efficacy of CDDP as a potent anticancer agent for GC [18, 24, 25]. They showed that CDDP significantly increased apoptosis in a concentration-dependent manner, in which AGS cells was more sensitive while NCI-N87 cells were more resistant to CDDP, consistent with our findings.. The NCI-N87 cells are HER-2 positive, and a previous study suggested that this may make cells more resistant to CDDP [26].

Studies have demonstrated that CDDP induces upregulation of p53 in GC cells, a finding consistent with our current study [27, 28]. On the other hand, CDDP reduced phosphorylation level of Akt, and hence its expression in GC cells [29], supporting our results where CDDP reduced Akt expression in the tested cells.

Whereas very few studies tested the efficacy of AKBA in cancer cells. Liu, et al. showed that AKBA at dose of 100 μM triggers apoptosis via a caspase-8-dependent pathway in colon cancer HT-29 cell line [17]. Another study showed that AKBA can inactivate STAT3 pathway, a pathway that participates in the activation of Akt gene expression [14]. Altogether, suggesting that AKBA can act on different molecular mechanisms and pathways accountable for inducing cell apoptosis in different cancer cell types. Examples of these targets at the cellular molecular level includes but not restricted to kinases, growth factors, transcription factors, enzymes and receptors [14]. Takada et al. detected that AKBA affects NFkB expression in lung adenocarcinoma H1299 cells and human T-cell leukemia Jurkat cells [30]. Moreover, the cytotoxic and antitumor effects are mainly due to induction of apoptosis through caspase activation, increased Bax expression, NF-κB downregulation and induction of poly (ADP)-ribosepolymerase (PARP) cleavage [30].

Although some studies have reported that AKBA appear to be a promising anticancer drug independently, studies in combination with clinically used anticancer drugs may suggest even more promising anti-cancer activity [16]. For example, Abdelaziz, et al. reported a potential anti-angiogenic effect of a combination of AKBA and CDDP against chemically-induced colon cancer (CC) in mice [21]. They transplanted CC cells in seventy healthy male albino mice and then treated them with AKBA, CDDP or both. Significant decrease in tumor proliferation index and serum markers was detected in the combination therapy arm rather than the mono-therapy, suggesting that AKBA augments the antitumor effect of CDDP.

## Conclusion

In conclusion, to the best of our knowledge, we are the first to show the effectiveness of AKBA in sensitizing gastric cancer cells to CDDP-induced apoptosis via p53-dependent pathway in GC cells. Understanding the molecular mechanisms of CDDP resistance and how to effectively overcome the resistance will provide insight in overcoming chemoresistance. One method of overcoming the ressitance is to use an adjuvant compound like AKBA, which itself has promising anticancer properties.

## Data Availability

Data sharing is not applicable to this article as no datasets were generated or analysed during the current study.

## Abbreviations

AKBA: Acetyl-11-Keto-Beta-Boswellic Acid
Bcl-2: B-Cell Lymphoma 2
CDDP: Cis–Diammine Dichloro Platinum
CO2: Carbon Dioxide
DNA: Deoxyribonucleic acid
FASL: Fatty Acid Synthase Ligand
GC: Gastric cancer
HER2: Human Epidermal Growth Factor Receptor 2
NFkB: Nuclear factor kappa-light-chain-enhancer of activated B cells
siRNA: small interference RNA
TNF: Tumor Necrosis Factor
